# Axillary traction: An effective method of resolving shoulder dystocia

**DOI:** 10.1111/ajo.13029

**Published:** 2019-07-10

**Authors:** Lesley Ansell, David Alan Ansell, Judith McAra‐Couper, Peter John Larmer, Nicholas Kenneth Gerald Garrett

**Affiliations:** ^1^ Birthing and Assessment Middlemore Hospital Auckland New Zealand; ^2^ Women's Health Middlemore Hospital Auckland New Zealand; ^3^ Faculty of Health and Environmental Sciences Auckland University of Technology Auckland New Zealand; ^4^ Faculty of Health and Environmental Sciences School of Clinical Sciences Auckland University of Technology Auckland New Zealand; ^5^ Faculty of Health and Environmental Sciences Department of Biostatistics and Epidemiology Auckland University of Technology Auckland New Zealand

**Keywords:** axillary traction, birth injury, posterior arm delivery, rotational manoeuvres, shoulder dystocia

## Abstract

**Background:**

At Counties Manukau Health in Auckland, New Zealand, axillary traction is being used when an internal manoeuvre is required for resolution of shoulder dystocia.

**Aims:**

This study presents the outcomes for mother and baby from use of axillary traction and other internal manoeuvres.

**Materials and Methods:**

Retrospective review of the clinical records of mother and baby for all labours complicated by shoulder dystocia was carried out for an eight‐year period. Maternal and neonatal information were compared for the three cohorts of the first internal manoeuvre documented: axillary traction, posterior arm delivery and rotational manoeuvres.

**Results:**

There were 226 women who required the use of internal manoeuvres with no significant differences in age, body mass index, parity, ethnicity, diabetes incidence, induction and augmentation of labour rates, length of the first stage and birth weight between the cohorts. Axillary traction was the first internal manoeuvre used for 119 (52.7%) with a success rate of 95.8%. Posterior arm delivery was used first for 49 (21.7%) women with a success rate of 85.7%. Rotational manoeuvres were used first for 58 (25.7%) women with a statistically inferior success rate of 48.3%. There was no significant difference in the maternal and neonatal complication rates between the cohorts.

**Conclusion:**

Axillary traction has been utilised as the first internal manoeuvre for a large number of women with a higher success rate than other internal manoeuvres without any increase in maternal or neonatal morbidity. It is recommended that this be the first internal manoeuvre attempted when shoulder dystocia occurs.

## Introduction

Shoulder dystocia is a childbirth emergency which has significant risks to fetal and maternal outcome.^1^ Shoulder dystocia occurs when either one or both shoulders fail to enter the pelvic cavity.^2^

Current management of shoulder dystocia involves the use of various manoeuvres to alleviate the problem, yet there is a lack of randomised controlled trials or experiments that have directly compared their effectiveness. There are authors who recommend a well‐coordinated sequence of manoeuvres such as those described by the HELPERR mnemonic,^3^, ^4^, ^5^ but there is no clear evidence base for the order of use of these manoeuvres.^6^, ^7^

**Figure 1:**
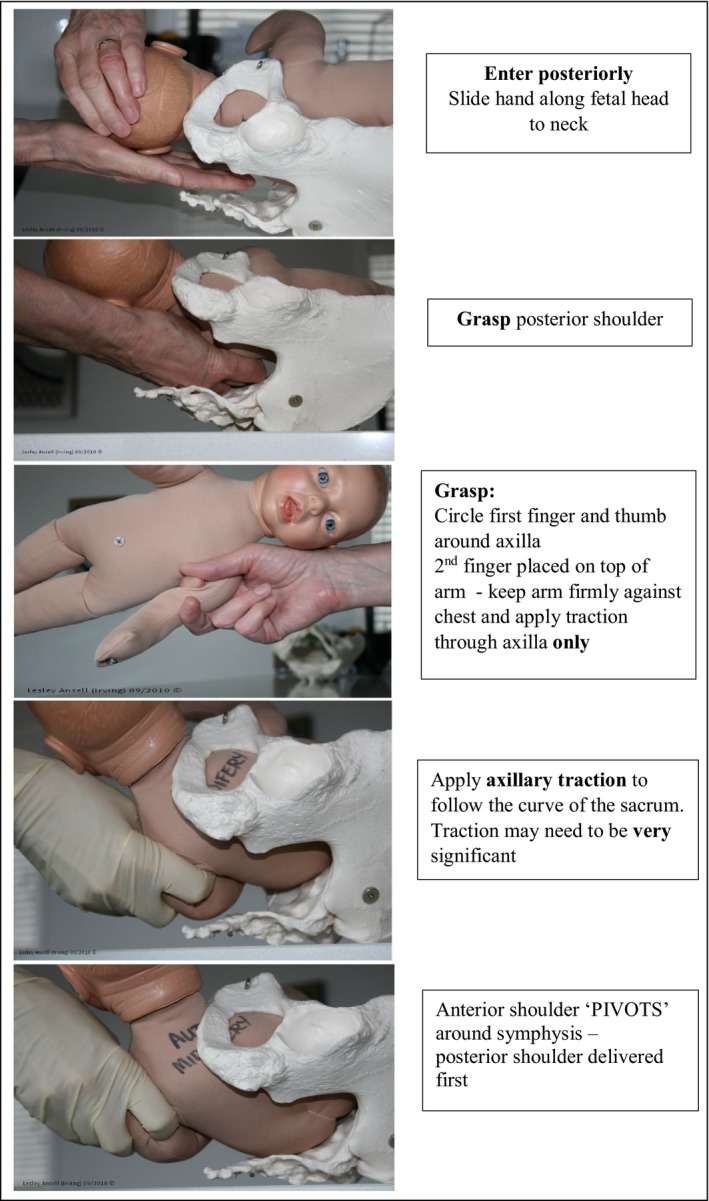
Axillary traction.

A qualitative study carried out by Ansell *et al*.^8^ suggested that axillary traction is a useful manoeuvre for the management of shoulder dystocia when an internal manoeuvre is required. To perform axillary traction the clinician's whole hand enters the posterior aspect of the pelvis. Regardless of which side the fetus is facing, the fetal shoulder is located and grasped by sliding the first finger under the axilla and placing the thumb on top of the shoulder. The second finger is placed alongside the fetal humerus to keep the arm firmly against the body. Traction is applied firmly and directly through the fetal axilla to follow the sacral curve until the posterior shoulder appears over the perineum while the anterior shoulder ‘pivots’ around the symphysis. Once the posterior shoulder is delivered then the anterior shoulder can easily be delivered by lateral traction (Fig. 1). This manoeuvre differs from removal of the posterior arm as only the axilla is located rather than the elbow. No attempt is made to flex the fetal arm across the body, the fetal arm is held firmly against the body and no traction or pressure applied to the humerus or elbow. The aim is to deliver the posterior shoulder only rather than the whole arm.

The aim of this study was to assess the effectiveness of axillary traction when shoulder dystocia occurs. This retrospective review of the internal manoeuvres used in the management of shoulder dystocia is the first to document the use of axillary traction for a large number of women.

## Materials and Methods

A retrospective review of clinical records of women who experienced shoulder dystocia between 1 January 2006 and 31 December 2013 was conducted. All women gave birth within Counties Manukau Health (CMH), Auckland. Participants were identified from the hospital database Casemix.

The study was approved by the Southern Health and Disability Ethics Committee (HDEC); Ethics reference: 14/STH/15.

The inclusion criteria for the study were women with a singleton fetus, cephalic presentation beyond 34 weeks gestation and who experienced shoulder dystocia during vaginal birth. Women with intrauterine fetal death before the onset of labour and major fetal abnormality were excluded.

Shoulder dystocia was defined as those women requiring more than the normal traction usually required or additional manoeuvres to effect delivery of the shoulders. There were a total of 52 055 vaginal births during the study period. A total of 422 (0.81%) were identified as having shoulder dystocia.

The maternal and neonatal records of all women experiencing shoulder dystocia during the study period were reviewed by the lead author and an assistant. Information regarding the manoeuvres used to resolve the dystocia and the order in which the manoeuvres were employed were collected. McRoberts and suprapubic pressure were treated as a single manoeuvre and in CMH these manoeuvres are universally employed first. The final successful manoeuvre was identified.

There were three main groups of internal manoeuvres:

1. axillary traction which included all manoeuvres documented as axillary traction or removal of the posterior shoulder
2. posterior arm delivery which was documented as such
3. internal rotational manoeuvres which included all manoeuvres documented as Woods' screw, reverse Woods' screw and/or internal rotation.

Maternal information included age, parity, ethnicity, body mass index (BMI), gestation, diabetes, induction of labour, augmentation of labour, epidural, normal vaginal birth, instrumental birth and type, length of the first stage and second stage of labour perineal trauma and blood loss. Neonatal information included birthweight, APGAR score < 7 at five minutes, brachial plexus injury (BPI), clavicular and humeral fractures, hypoxic ischaemic encephalopathy (HIE) and neonatal unit admission. Cord blood gas analysis results were not readily available during the study period; therefore, they were unable to be collected.

Data were collected onto a Microsoft Excel spreadsheet which was then analysed using the Statistical Package for the Social Sciences (SPSS) version 24 (IBM, Armonk, NY, USA). Demographic and clinical data for the three internal manoeuvre cohorts were compared. Categorical data were compared using χ^2^ and Fisher's exact tests. Continuous data were compared using Student's *t*‐test (normal distribution) or Mann–Whitney or Kruskall–Wallace test for non‐normal distributions. Where differences between the cohorts were noted with categorical data the success rates of the internal manoeuvres were compared using χ^2^ tests or Fisher's exact test.

## Results

Data were collected for the 422 women whose births were complicated by shoulder dystocia at CMH for the period 1 January 2006 to 31 December 2013 (inclusive). The incidence of shoulder dystocia increased from 0.54% of all vaginal births in 2006 to 1.26% in 2013 with the average rate of shoulder dystocia over the study period being 0.81%.

Of the 422 women who experienced shoulder dystocia, a total of 226 required internal manoeuvres to resolve the problem (53.6%). The first internal manoeuvre used was as follows:

1. axillary traction in 119 women (52.7%)
2. posterior arm delivery in 49 women (21.7%)
3. rotational manoeuvres in 58 women (25.7%).

There were no significant differences in age, BMI, parity, gestation, ethnicity, incidence of diabetes, labour induction rates, syntocinon augmentation rates, epidural use, length of the first stage, and birth weight in the three cohorts (Table 1). The axillary traction cohort was less often preceded by ventouse than the posterior arm delivery and rotational manoeuvres cohort (*P* 0.03). The overall success rate of the first internal manoeuvre was no different if the ventouse was used or not (*P* 0.56). Labour induction rates between the cohorts neared statistical significance (*P* 0.07) but the overall success rate of the first internal manoeuvre was no different if it was preceded by labour induction (χ^2^ *P* 0.59). Only 13 women had forceps deliveries with no statistically significant difference between the cohorts (0.13) and the overall success rate of the first internal manoeuvre was no different if it was preceded by forceps delivery (*P* 0.71). Length of the second stage of labour was significantly different between the cohorts (*P* 0.03) with the posterior arm delivery cohort having a median second stage 26 min longer than the axillary traction cohort and 18.5 min longer than the rotational manoeuvres cohort. Again, the distribution of the length of the second stage was no different if the first internal manoeuvre was successful or not (*P* 0.25). Median length of the first stage was not significantly different for the three cohorts (*P* 0.19).

**Table 1:** Demographics and clinical characteristics of first internal manoeuvre cohorts

	Axillary traction	Posterior arm delivery	Rotational manoeuvres	*P‐*value
Characteristics				
Age, years, median	29.0	31.0	28.0	0.26†
Body mass index, median	30.2	28.1	31.8	0.70†
Parity				
Nulliparous	35 (29.4%)	19 (38.8%)	18 (31.0%)	0.49
Multiparous	84 (70.6%)	30 (61.2%)	40 (69.0%)	
Gestation, weeks, median	40.1	40.1	40.0	0.50†
Ethnicity				
European	22 (18.5%)	14 (28.6%)	9 (15.6%)	0.15
Māori	10 (8.4%)	5 (10.2%)	11 (19.0%)	
Pacifica	44 (37.0%)	9 (18.4%)	20 (34.5%)	
Indian	21 (17.6%)	13 (26.5%)	9 (15.5%)	
Chinese	5 (4.2%)	4 (8.2%)	3 (5.2%)	
Other	17 (14.3%)	4 (8.2%)	6 (10.3%)	
Diabetes				
Type 1	0	1 (2.0%)	0	0.40‡
Type 2	2 (1.7%)	2 (4.1%)	3 (5.2%)	
Gestational diabetes	11 (9.2%)	6 (12.2%)	7 (12.1%)	
Labour characteristics				
Labour induction	21 (17.6%)	16 (32.7%)	10 (17.2%)	0.07
Syntocinon augmentation	34 (28.6%)	19 (38.8%)	15 (25.9%)	0.30
Epidural	25 (21.0%)	14 (29.2%)	15 (25.9%)	0.50
Forceps delivery	5 (4.2%)	6 (12.2%)	2 (3.4%)	0.13‡
Ventouse delivery	23 (19.3%)	20 (40.8%)	16 (27.6%)	0.03
Length of first stage, h, median	5.875	6.0	6.75	0.19†
Length of second stage, min, median	33.0	59.0	40.5	0.03†
Birth weight, g, median	4060	4080	4090	0.77†

† Kruskall–Wallace test.

‡ Fisher exact test.

Overall there was a highly significant difference (*P* < 0.001) in the success rates of the first used internal manoeuvres (Table 2). In 119 (52.7%) cases the first internal manoeuvre used was axillary traction. This was successful in 114 cases (95.8%) and no further manoeuvres were required. Of the five which failed with axillary traction as the first internal manoeuvre, three were successfully delivered with the second use of axillary traction by a different practitioner, one with rotational manoeuvres and one with rotational manoeuvre followed by posterior arm delivery. The success rate of axillary traction as a first internal manoeuvre was significantly greater than that for rotational manoeuvres (*P* < 0.001) and posterior arm delivery (*P* 0.025).

**Table 2:** Success rates of the first three internal manoeuvres used

	Success *n* (%)	Failure *n* (%)	Total *n*	*P*‐value
First manoeuvre				
Axillary traction	114 (95.8)	5 (4.2)	119	<0.001
Posterior arm	42 (85.7)	7 (14.3)	49	
Internal rotation	28 (48.3)	30 (51.7)	58	
Total	184 (81.4)	42 (18.6)	226	
Second manoeuvre				
Axillary traction	12 (85.7)	2 (14.3)	14	0.71†
Posterior arm	11 (78.6)	3 (21.4)	14	
Internal rotation	7 (70.0)	3 (30.0)	10	
Total	30 (78.9)	8 (21.1)	38	
Third manoeuvre				
Axillary traction	4	1	5	0.52†
Posterior arm	1	0	1	
Internal rotation	0	1	1	
Total	5	2	7	

† Fisher exact test.

In 49 (21.7%) cases posterior arm delivery was used as the first internal manoeuvre and was successful in 42 (85.7%) cases. In the seven cases where posterior arm delivery failed, one was delivered with a second attempt of the posterior arm, four with rotational manoeuvres and two with axillary traction. The success rate of use of posterior arm delivery was significantly less than that of use of axillary traction (*P* 0.025) but much higher than that of rotational manoeuvres (*P* < 0.01).

In 58 (25.7%) cases, a rotational manoeuvre was the first internal manoeuvre attempted and was successful in 28 cases (48.3%) which is statistically less successful than axillary traction (*P* < 0.001) and posterior arm delivery (*P* < 0.01). Of the 28 cases where rotational manoeuvre was unsuccessful as the first manoeuvre, nine were delivered by axillary traction, 10 with posterior arm delivery, three with a further attempt at rotational manoeuvres and six were delivered with a combination of other manoeuvres.

Axillary traction was used as a second internal manoeuvre for 14 women (Table 2), posterior arm delivery as a second manoeuvre for 14 women and rotational manoeuvres for 10 women. Overall there was no significant difference in the success rates of the different second manoeuvres (*P* 0.71).

A third internal manoeuvre was required for just seven women with again there being no significant difference in success rates (*P* 0.52).

There were no significant differences in neonatal and maternal complication rates between those managed without internal manoeuvres and those requiring any internal manoeuvres except for BPI. All BPI were Erb's palsies and all but five had recovered before hospital discharge. Axillary traction had been used for only one of these babies and all had recovered completely by three months of age (Table 3).

**Table 3:** Complications with and without internal manoeuvres

	No internal manoeuvres	Any internal manoeuvres	*P*‐value
Neonatal			
APGAR < 7 at 5 min	8 (4.1%)	16 (7.1%)	0.18
Brachial plexus injury	15 (7.7%)	37 (16.4%)	0.02
Clavicle fracture	3 (1.5%)	3 (1.3%)	0.72†
Humerus fracture	1 (0.5%)	5 (2.2%)	0.22†
Hypoxic ischaemic encephalopathy			
Grade 1	0	2	0.28†
Grade 2	0	0	
Grade 3	1	1	
Neonatal unit admission	22 (11.3%)	34 (15.0%)	0.26
Maternal			
Blood loss, mL			0.90‡
Mean	477	458	0.90
Perineum			
Episiotomy	41 (21.1%)	63 (28.8%)	
Second degree laceration	57 (29.4%)	51 (23.3%)	0.35
Third/fourth degree laceration	18 (9.3%)	16 (7.3%)	

† Fisher exact test.

‡ Kruskall–Wallace test.

There were no significant differences in the complication rates (Table 4) of the different first manoeuvres in relation to perineal trauma (*P* 0.36), total neonatal birth injuries (*P* 0.39) and neonatal nerve palsies (*P* 0.70). There were just three recorded cases with HIE.

**Table 4:** Complications associated with internal manoeuvres

	Axillary traction	Posterior arm delivery	Rotational manoeuvres	*P‐*value
Neonatal				
APGAR < 7 at 5 min	1 (0.8%)	3 (6.1%)	12 (20.1%)	<0.001†
Brachial plexus injury	17 (14.3%)	10 (20.4%)	10 (17.2%)	0.70
Clavicular fracture	3 (2.5%)	0	0	0.15†
Humerus fracture	1 (0.8%)	1 (2.0%)	3 (5.3%)	0.21†
Hypoxic ischaemic encephalopathy				
Grade 1	2	0	0	0.59†
Grade 2	0	0	0	
Grade 3	0	0	1	
NNU admission	18 (15.1%)	6 (12.2%)	10 (17.2%)	0.7
Maternal				
Blood loss (mL)				
Mean	373	560	546	0.03‡
Perineum				
Episiotomy	28 (22.2%)	21 (43.8%)	14 (24.1%)	0.36†
Second degree tear	24 (20.9%)	13 (22.9%)	16 (27.6%)	
Third/fourth degree tear	9 (7.8%)	3 (6.3%)	4 (6.9%)	

† Fisher exact test.

‡ Kruskall–Wallace test.

NNU, neonatal unit

## Discussion

Management of shoulder dystocia involves the use of both internal and external manoeuvres to overcome the problem, yet there is a lack of randomised controlled trials (RCT) to compare their effectiveness. Such trials would not be feasible because of the difficulty in obtaining informed consent from all women for different manoeuvres when managing an uncommon complication of vaginal birth and every measure possible to resolve the problem is required without the restriction of one defined set of manoeuvres as would be required with a RCT. The internal manoeuvres currently used to resolve shoulder dystocia, therefore, have been implemented largely because of case reports, individual practitioner experience and expert opinion.^9^, ^10^

MacKenzie *et al*.^11^ report increasing rates of shoulder dystocia with a trend of 0.3% per year. The rates of shoulder dystocia in the study were seen to increase from 0.54% in 2006 to 1.26% in 2013. This is an increasing trend of 0.12% per year, comparable with the study of MacKenzie *et al*.^11^

The average BMI in the study population was 30.87 which is categorised as obese^12^ and there is evidence that maternal obesity and fetal macrosomia is associated with shoulder dystocia.^13^, ^14^

The results of this study show that axillary traction was a highly successful manoeuvre when used as the first internal manoeuvre (96.4%) and no further manoeuvres were required. Posterior arm delivery also had a significant success rate (84.8%) when used as the first internal manoeuvre but was not as successful as axillary traction. Posterior arm delivery was significantly more successful than rotational manoeuvres (46.6%) but neither manoeuvre was as successful as axillary traction.

The choice of the first internal manoeuvre used was not affected by ethnicity, maternal age, BMI, maternal diabetes, gestation, labour induction, syntocinon augmentation, epidural, length of the first stage, forceps delivery or birthweight. Posterior arm delivery was significantly more likely to be the first manoeuvre used in women who had a longer second stage or ventouse delivery.

Shoulder dystocia is associated with birth injuries such as BPI, skeletal fractures, birth asphyxia and neurological injury.^1^, ^15^ It is likely that the higher rate of BPI seen in the group managed by internal manoeuvres was due to the use of multiple manoeuvres and/or the severity of the shoulder dystocia.

In this study, axillary traction had been used for a large number of women with no evidence of increased adverse effects on the neonate. There was a slightly lower incidence of birth injuries in the axillary traction group, but this was not statistically significant. There were no statistical differences in the number of Erb's palsies and total birth injuries in any of the groups. From this case series, there is no indication that axillary traction increases the risk of BPI. Shoulder dystocia is associated with higher rates of BPI^16^ and is possibly related to excessive traction on the fetal head rather than the internal manoeuvres used to resolve the problem.^17^ The amount of traction applied to the fetal head in all cases in the study population was unable to be assessed due to lack of documentation.

The results of this study show that axillary traction is a very effective manoeuvre for resolving shoulder dystocia with no increased adverse outcomes to the neonate and should be considered as the first‐line management when an internal manoeuvre is required.

### Benefits and limitations of the study

One of the main benefits of this study is that there were multiple ethnicities and high average BMI in the study population which means that the results are applicable to most women.

The limitations of the study are acknowledged. There was no consistency in the definition of shoulder dystocia and diagnosis was based on clinical judgement. However, as the cases of shoulder dystocia in the study population all required an internal manoeuvre to resolve the problem, there is an assumption that the shoulder dystocia was significant. The head‐to‐body delivery time intervals were seldom recorded so this measure, often used in diagnosis and assessing severity of shoulder dystocia^18^ is not available. However, this measure of severity may not be applicable to this study population as it is usual practice to wait for the next contraction following birth of the head before attempting delivery of the shoulders.

Data collection was difficult because there was no formal method for documenting shoulder dystocia. It is possible that not all of the manoeuvres used may have been recorded, with only the manoeuvre that resolved the shoulder dystocia being recorded. Even when a proforma was introduced in 2010 to capture the order of the manoeuvres used and the length of time each manoeuvre was attempted, it was often not completed and methods of management were extracted from the written clinical notes.

It is also unclear as to why the practitioner chose a particular manoeuvre as there were no clinical criteria as to when to use each manoeuvre. This may be related to individual practitioners who manage shoulder dystocia depending on their training and experience or to the perceived difficulty of the shoulder dystocia. Resident Medical Officers who had worked in other hospitals may not have been exposed to axillary traction and may, therefore, have been more likely to use delivery of the posterior arm or rotational manoeuvres.

## Conclusion

This retrospective study showed a significant increase in the rate of shoulder dystocia for women in the study period (0.54–1.26%). The reason for this increase is unknown but may be related to the high level of diabetes and obesity in the study population.

Axillary traction is found to be a highly successful manoeuvre when used as the first internal manoeuvre (95.8%). Removal of the posterior arm also had a significant success rate (84.8%) when used as the first internal manoeuvre but was not as successful as axillary traction but significantly more successful than internal rotation. There were slightly less birth injuries in axillary traction group and although not statistically significant warrants further investigation.

This study, therefore, provides good evidence that axillary traction has a high success rate and a low complication rate and can be used for all women. Axillary traction should be recommended as the first internal manoeuvre attempted when shoulder dystocia occurs.
